# (5*S*)-3-Chloro-4-diallyl­amino-5-[(1*R*,2*S*,5*R*)-2-isopropyl-5-methyl­cyclo­hex­yloxy]furan-2(5*H*)-one

**DOI:** 10.1107/S1600536811027772

**Published:** 2011-07-16

**Authors:** Dong-Na Huang, Yue-He Tan, Jian-Hua Fu, Zhao-Yang Wang

**Affiliations:** a School of Chemistry and Environment, South China Normal University, Guangzhou 510006, People’s Republic of China

## Abstract

The title compound, C_20_H_30_ClNO_3_, was obtained *via* a tandem asymmetric Michael addition–elimination reaction of (5*S*)-3,4-dichloro-5-(*l*-menth­yloxy)-2(5*H*)-furan­one and diallyl­amine in the presence of potassium fluoride. The mol­ecular structure contains an approximately planar five-membered furan­one ring [maximum atomic deviation = 0.0221 (3) Å] and a six-membered ring adopting a chair conformation.

## Related literature

For the biological activity of 4-amino-2(5*H*)-furan­ones, see: Gondela & Walczak (2010 ▶). For chemical, pharmaceutical and agrochemical applications of 3,4-amino-2(5*H*)-furan­ones, see: Tanoury *et al.* (2008 ▶); Kimura *et al.* (2000 ▶). For the synthesis of optically pure 5-(*l*-menth­yloxy)-3,4-dichloro-2(5*H*)-furan­ones, see: Song *et al.* (2009 ▶). For the use of inter­mediate chiral 5-*S*-(*l*-menth­yloxy)-2(5*H*)-furan­ones, see: Hoffmann *et al.* (2006 ▶).
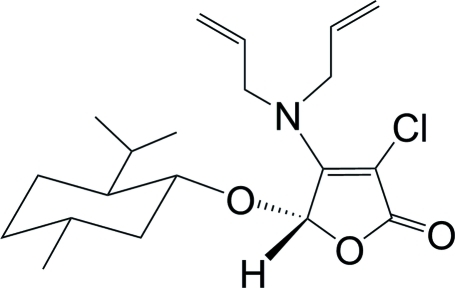


## Experimental

### 

#### Crystal data

C_20_H_30_ClNO_3_
*M* *_r_* = 367.90
Orthorhombic, 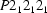

*a* = 8.4540 (17) Å
*b* = 11.722 (2) Å
*c* = 20.648 (4) Å
*V* = 2046.1 (7) Å^3^
*Z* = 4
Mo *K*α radiation
μ = 0.20 mm^−1^
*T* = 273 K
0.23 × 0.20 × 0.16 mm

#### Data collection

Bruker APEXII CCD area-detector diffractometer
Absorption correction: multi-scan (*SADABS*; Sheldrick, 1996 ▶) *T*_min_ = 0.954, *T*_max_ = 0.968
9931 measured reflections
3950 independent reflections
2923 reflections with *I* > 2σ(*I*)
*R*_int_ = 0.033

#### Refinement

*R*[*F* ^2^ > 2σ(*F* ^2^)] = 0.038
*wR*(*F* ^2^) = 0.102
*S* = 0.97
3950 reflections
230 parameters
H-atom parameters constrained
Δρ_max_ = 0.11 e Å^−3^
Δρ_min_ = −0.13 e Å^−3^
Absolute structure: Flack (1983 ▶), 1677 Friedel pairs
Flack parameter: −0.06 (6)

### 

Data collection: *APEX2* (Bruker, 2008 ▶); cell refinement: *SAINT* (Bruker, 2008 ▶); data reduction: *SAINT*; program(s) used to solve structure: *SHELXS97* (Sheldrick, 2008 ▶); program(s) used to refine structure: *SHELXL97* (Sheldrick, 2008 ▶); molecular graphics: *ORTEP-3 for Windows* (Farrugia, 1997 ▶); software used to prepare material for publication: *SHELXL97*.

## Supplementary Material

Crystal structure: contains datablock(s) global, I. DOI: 10.1107/S1600536811027772/zq2113sup1.cif

Structure factors: contains datablock(s) I. DOI: 10.1107/S1600536811027772/zq2113Isup2.hkl

Supplementary material file. DOI: 10.1107/S1600536811027772/zq2113Isup3.cml

Additional supplementary materials:  crystallographic information; 3D view; checkCIF report

## Figures and Tables

**Fig. 1.**
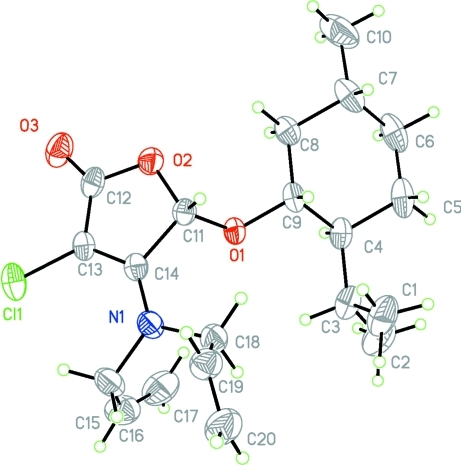
The molecular structure of the title compound showing thermal ellipsoids drawn at the 50% probability level.

**Fig. 2.**
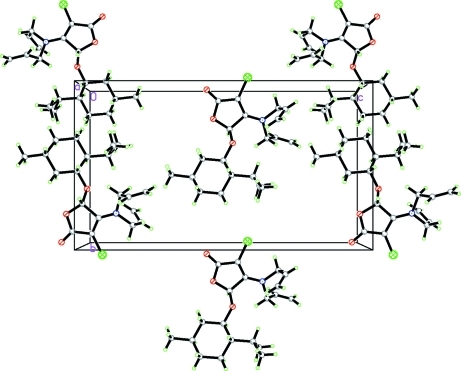
Perspective view of the crystal packing.

## supplementary crystallographic information

### Comment

The 2(5*H*)-furanone moiety is present in many natural products exhibiting various biological activities, namely antibiotic cytotoxic and antitumor (Gondela & Walczak, 2010). Recently, owing to their specific activity and high stereoselectivity, chiral 5*S*-(*l*-menthyloxy)-2(5*H*)-furanones have emerged as significant synthetic intermediates (Hoffmann *et al.*, 2006; Song *et al.*, 2009). At the same time, 4-amino-2(5*H*)-furanone (or 3-amino-2(5*H*)-furanone) is a kind of attractive moiety in chemical, pharmaceutical and agrochemical research (Tanoury *et al.*, 2008; Kimura *et al.*, 2000). Therefore we were interested in the tandem Michael addition-elimination reaction of the chiral synthon 3,4-dichloro-5(*S*)-(*l*-menthyloxy)-2(5*H*)-furanone and diallylamine in the present of potassium fluoride which yielded the title compound, C_20_H_30_ClNO_3_, illustrated in Fig. 1.

The title compound has four chiral centers [C4(*S*), C7(*R*), C9(*R*), C11(*S*)] and contains a five-membered furanone ring and a six-membered ring connected to each other *via* a C11—O1—C9 ether bond. The furanone ring of C11—O2—C12—C13—C14 is approximately planar [maximum atomic deviation 0.0221 (3) Å], whereas the six-membered ring displays a chair conformation.

### Experimental

The precursor 3,4-dichloro-5-(*S*)-(*l*-menthyloxy)-2(5*H*)-furanone was prepared according to the literature procedure (Song *et al.*, 2009). After the mixture of 3,4-dichloro-5*S*-(*l*-menthyloxy)-2(5*H*)-furanone (2.0 mmol) and potassium fluoride (6.0 mmol) was dissolved in absolute tetrahydrofuran (2.0 ml) under nitrogen atmosphere, tetrahydrofuran solution of diallylamine (3.0 mmol) was added. The reaction was carried out under the stirring at room temperature for 24 h. Once the reaction was complete, the solvents were removed under reduced pressure. The residual solid was dissolved in dichloromethane. Then the combined organic layers from extraction were concentrated under reduced pressure, and the crude product was purified by silica gel column chromatography with the gradient mixture of petroleum ether and ethyl acetate to give the final product (0.575 g, 78.3%).

### Refinement

All H atoms were positioned in calculated positions and refined using a riding model, with C—H = 0.93 Å and *U*_iso_(H) = 1.2 *U*_eq_(C) for aromatic H atoms, with C—H = 0.97 Å and *U*_iso_(H) = 1.2 *U*_eq_(C) for methylene H atoms, with C—H = 0.98 Å and *U*_iso_(H) = 1.2 *U*_eq_(C) for methine H atoms, and with C—H = 0.96 Å and *U*_iso_(H) = 1.5 *U*_eq_(C) for methyl H atoms.

### Crystal data

**Table:**

C_20_H_30_ClNO_3_	*F*(000) = 792.0
*M**_r_* = 367.90	*D*_x_ = 1.194 Mg m^−^^3^
Orthorhombic, *P*2_1_2_1_2_1_	Mo *K*α radiation, λ = 0.71073 Å
Hall symbol: P 2ac 2ab	Cell parameters from 2994 reflections
*a* = 8.4540 (17) Å	θ = 2.6–22.1°
*b* = 11.722 (2) Å	µ = 0.20 mm^−^^1^
*c* = 20.648 (4) Å	*T* = 273 K
*V* = 2046.1 (7) Å^3^	Block, colourless
*Z* = 4	0.23 × 0.20 × 0.16 mm

### Data collection

**Table:**

Bruker APEXII CCD area-detector diffractometer	3950 independent reflections
Radiation source: fine-focus sealed tube	2923 reflections with *I* > 2σ(*I*)
graphite	*R*_int_ = 0.033
phi and ω scans	θ_max_ = 26.0°, θ_min_ = 2.0°
Absorption correction: multi-scan (*SADABS*; Sheldrick, 1996)	*h* = −10→8
*T*_min_ = 0.954, *T*_max_ = 0.968	*k* = −14→14
9931 measured reflections	*l* = −22→25

### Refinement

**Table:**

Refinement on *F*^2^	Hydrogen site location: inferred from neighbouring sites
Least-squares matrix: full	H-atom parameters constrained
*R*[*F*^2^ > 2σ(*F*^2^)] = 0.038	*w* = 1/[σ^2^(*F*_o_^2^) + (0.0584*P*)^2^] where *P* = (*F*_o_^2^ + 2*F*_c_^2^)/3
*wR*(*F*^2^) = 0.102	(Δ/σ)_max_ < 0.001
*S* = 0.97	Δρ_max_ = 0.11 e Å^−^^3^
3950 reflections	Δρ_min_ = −0.13 e Å^−^^3^
230 parameters	Extinction correction: *SHELXL97* (Sheldrick, 2008), Fc^*^=kFc[1+0.001xFc^2^λ^3^/sin(2θ)]^-1/4^
0 restraints	Extinction coefficient: 0.0125 (18)
Primary atom site location: structure-invariant direct methods	Absolute structure: Flack (1983), 1677 Friedel pairs
Secondary atom site location: difference Fourier map	Flack parameter: −0.06 (6)

### Special details

**Table:**

Geometry. All e.s.d.'s (except the e.s.d. in the dihedral angle between two l.s. planes) are estimated using the full covariance matrix. The cell e.s.d.'s are taken into account individually in the estimation of e.s.d.'s in distances, angles and torsion angles; correlations between e.s.d.'s in cell parameters are only used when they are defined by crystal symmetry. An approximate (isotropic) treatment of cell e.s.d.'s is used for estimating e.s.d.'s involving l.s. planes.
Refinement. Refinement of *F*^2^ against ALL reflections. The weighted *R*-factor *wR* and goodness of fit *S* are based on *F*^2^, conventional *R*-factors *R* are based on *F*, with *F* set to zero for negative *F*^2^. The threshold expression of *F*^2^ > σ(*F*^2^) is used only for calculating *R*-factors(gt) *etc*. and is not relevant to the choice of reflections for refinement. *R*-factors based on *F*^2^ are statistically about twice as large as those based on *F*, and *R*- factors based on ALL data will be even larger.

### Fractional atomic coordinates and isotropic or equivalent isotropic displacement parameters (Å^2^)

**Table:**

	*x*	*y*	*z*	*U*_iso_*/*U*_eq_	
Cl1	0.27814 (9)	0.46092 (4)	0.91712 (3)	0.0767 (2)	
C14	0.3852 (2)	0.68664 (15)	0.92440 (10)	0.0450 (5)	
C12	0.3084 (3)	0.60235 (16)	1.01887 (11)	0.0553 (5)	
C13	0.3270 (3)	0.58965 (15)	0.95061 (10)	0.0513 (5)	
C11	0.3995 (3)	0.77169 (15)	0.97949 (9)	0.0455 (5)	
H11	0.5078	0.8009	0.9827	0.055*	
C4	0.2172 (3)	1.05546 (15)	0.97288 (11)	0.0560 (5)	
H4	0.1096	1.0295	0.9827	0.067*	
C8	0.3116 (3)	0.95600 (17)	1.07345 (10)	0.0595 (6)	
H8A	0.3896	0.9024	1.0895	0.071*	
H8B	0.2077	0.9252	1.0830	0.071*	
C9	0.3297 (3)	0.96703 (16)	1.00113 (9)	0.0496 (5)	
H9	0.4389	0.9890	0.9911	0.060*	
C6	0.2206 (4)	1.1564 (2)	1.08117 (14)	0.0844 (8)	
H6A	0.2396	1.2296	1.1017	0.101*	
H6B	0.1125	1.1341	1.0905	0.101*	
C19	0.6469 (3)	0.81529 (18)	0.81335 (11)	0.0645 (6)	
H19	0.7300	0.7852	0.8374	0.077*	
C5	0.2408 (4)	1.16881 (17)	1.00889 (14)	0.0805 (8)	
H5A	0.3461	1.1976	0.9999	0.097*	
H5B	0.1652	1.2242	0.9928	0.097*	
C18	0.4919 (3)	0.82587 (17)	0.84594 (11)	0.0591 (6)	
H18A	0.5042	0.8718	0.8847	0.071*	
H18B	0.4189	0.8652	0.8174	0.071*	
C20	0.6762 (4)	0.8440 (2)	0.75483 (13)	0.0879 (9)	
H20A	0.5963	0.8744	0.7291	0.105*	
H20B	0.7774	0.8345	0.7380	0.105*	
C15	0.3794 (3)	0.6432 (2)	0.80906 (11)	0.0696 (7)	
H15A	0.3966	0.5640	0.8207	0.083*	
H15B	0.4479	0.6606	0.7727	0.083*	
C3	0.2284 (3)	1.06627 (18)	0.89904 (12)	0.0692 (7)	
H3	0.2244	0.9886	0.8815	0.083*	
C2	0.0861 (4)	1.1300 (3)	0.87141 (16)	0.1021 (11)	
H2A	0.0887	1.1263	0.8250	0.153*	
H2B	0.0897	1.2083	0.8850	0.153*	
H2C	−0.0096	1.0954	0.8869	0.153*	
C7	0.3313 (3)	1.0696 (2)	1.10909 (12)	0.0737 (8)	
H7	0.4397	1.0964	1.1019	0.088*	
C17	0.1074 (4)	0.7219 (3)	0.81581 (15)	0.0960 (10)	
H17A	0.1338	0.7650	0.8521	0.115*	
H17B	0.0053	0.7251	0.7991	0.115*	
C16	0.2130 (5)	0.6574 (3)	0.78848 (13)	0.0877 (9)	
H16	0.1808	0.6161	0.7524	0.105*	
C1	0.3837 (4)	1.1195 (3)	0.87538 (15)	0.0915 (9)	
H1A	0.4714	1.0808	0.8952	0.137*	
H1B	0.3862	1.1988	0.8870	0.137*	
H1C	0.3908	1.1121	0.8292	0.137*	
C10	0.3083 (5)	1.0534 (3)	1.18160 (13)	0.1167 (12)	
H10A	0.2053	1.0221	1.1896	0.175*	
H10B	0.3177	1.1258	1.2030	0.175*	
H10C	0.3876	1.0023	1.1979	0.175*	
O1	0.29340 (17)	0.86000 (10)	0.96896 (6)	0.0459 (3)	
O3	0.2592 (2)	0.53592 (13)	1.05869 (8)	0.0787 (5)	
O2	0.3578 (2)	0.70923 (11)	1.03625 (6)	0.0578 (4)	
N1	0.4245 (2)	0.71508 (14)	0.86380 (8)	0.0535 (5)	

### Atomic displacement parameters (Å^2^)

**Table:**

	*U*^11^	*U*^22^	*U*^33^	*U*^12^	*U*^13^	*U*^23^
Cl1	0.0780 (5)	0.0447 (3)	0.1075 (5)	−0.0086 (3)	0.0051 (4)	−0.0110 (3)
C14	0.0359 (12)	0.0431 (10)	0.0560 (12)	0.0047 (8)	0.0009 (10)	−0.0023 (9)
C12	0.0476 (14)	0.0461 (11)	0.0721 (14)	0.0114 (10)	0.0083 (12)	0.0082 (10)
C13	0.0499 (14)	0.0391 (10)	0.0650 (13)	0.0052 (9)	0.0028 (11)	−0.0034 (9)
C11	0.0420 (13)	0.0424 (10)	0.0520 (11)	0.0048 (8)	0.0026 (10)	0.0002 (9)
C4	0.0468 (13)	0.0410 (10)	0.0802 (14)	0.0021 (9)	0.0065 (12)	0.0006 (9)
C8	0.0590 (15)	0.0544 (12)	0.0651 (13)	0.0027 (11)	0.0038 (12)	−0.0137 (10)
C9	0.0429 (12)	0.0384 (9)	0.0676 (13)	−0.0027 (9)	0.0066 (10)	−0.0081 (9)
C6	0.0707 (19)	0.0605 (13)	0.122 (2)	0.0036 (13)	0.0113 (19)	−0.0395 (14)
C19	0.0649 (17)	0.0676 (14)	0.0611 (14)	−0.0026 (11)	0.0068 (12)	0.0045 (11)
C5	0.0714 (18)	0.0416 (11)	0.128 (2)	0.0022 (11)	0.0092 (18)	−0.0092 (12)
C18	0.0696 (18)	0.0510 (12)	0.0567 (13)	−0.0041 (11)	0.0104 (12)	−0.0008 (9)
C20	0.102 (3)	0.0845 (18)	0.0771 (18)	0.0078 (16)	0.0255 (17)	0.0160 (13)
C15	0.085 (2)	0.0660 (14)	0.0577 (13)	−0.0072 (13)	0.0093 (14)	−0.0186 (11)
C3	0.0683 (18)	0.0532 (12)	0.0860 (17)	0.0052 (11)	0.0083 (14)	0.0154 (11)
C2	0.097 (3)	0.093 (2)	0.117 (2)	0.0256 (18)	−0.001 (2)	0.0334 (19)
C7	0.0587 (18)	0.0717 (15)	0.0907 (18)	−0.0038 (12)	0.0042 (14)	−0.0356 (13)
C17	0.072 (2)	0.112 (2)	0.103 (2)	0.0005 (18)	−0.0199 (19)	0.0284 (19)
C16	0.095 (3)	0.097 (2)	0.0713 (17)	−0.0248 (18)	−0.0220 (18)	0.0032 (14)
C1	0.091 (2)	0.0711 (17)	0.112 (2)	0.0002 (15)	0.0313 (18)	0.0273 (16)
C10	0.132 (3)	0.124 (2)	0.095 (2)	0.017 (2)	−0.009 (2)	−0.0571 (18)
O1	0.0451 (9)	0.0361 (6)	0.0566 (8)	0.0029 (6)	−0.0014 (7)	−0.0035 (5)
O3	0.0931 (14)	0.0606 (9)	0.0823 (11)	0.0075 (10)	0.0270 (10)	0.0186 (8)
O2	0.0713 (11)	0.0498 (8)	0.0524 (8)	0.0084 (7)	0.0055 (8)	0.0020 (6)
N1	0.0601 (13)	0.0492 (9)	0.0512 (10)	−0.0060 (8)	0.0077 (9)	−0.0074 (8)

### Geometric parameters (Å, °)

**Table:**

Cl1—C13	1.711 (2)	C5—H5B	0.9700
C14—N1	1.337 (3)	C18—N1	1.466 (3)
C14—C13	1.352 (3)	C18—H18A	0.9700
C14—C11	1.517 (3)	C18—H18B	0.9700
C12—O3	1.206 (2)	C20—H20A	0.9300
C12—O2	1.369 (2)	C20—H20B	0.9300
C12—C13	1.426 (3)	C15—N1	1.460 (3)
C11—O1	1.387 (2)	C15—C16	1.479 (4)
C11—O2	1.426 (2)	C15—H15A	0.9700
C11—H11	0.9800	C15—H15B	0.9700
C4—C9	1.523 (3)	C3—C2	1.526 (4)
C4—C3	1.533 (3)	C3—C1	1.534 (4)
C4—C5	1.536 (3)	C3—H3	0.9800
C4—H4	0.9800	C2—H2A	0.9600
C8—C9	1.507 (3)	C2—H2B	0.9600
C8—C7	1.530 (3)	C2—H2C	0.9600
C8—H8A	0.9700	C7—C10	1.521 (4)
C8—H8B	0.9700	C7—H7	0.9800
C9—O1	1.452 (2)	C17—C16	1.299 (4)
C9—H9	0.9800	C17—H17A	0.9300
C6—C7	1.498 (4)	C17—H17B	0.9300
C6—C5	1.509 (4)	C16—H16	0.9300
C6—H6A	0.9700	C1—H1A	0.9600
C6—H6B	0.9700	C1—H1B	0.9600
C19—C20	1.279 (3)	C1—H1C	0.9600
C19—C18	1.478 (4)	C10—H10A	0.9600
C19—H19	0.9300	C10—H10B	0.9600
C5—H5A	0.9700	C10—H10C	0.9600
			
N1—C14—C13	132.44 (19)	C19—C18—H18B	109.1
N1—C14—C11	121.21 (17)	H18A—C18—H18B	107.8
C13—C14—C11	106.34 (18)	C19—C20—H20A	120.0
O3—C12—O2	121.2 (2)	C19—C20—H20B	120.0
O3—C12—C13	130.1 (2)	H20A—C20—H20B	120.0
O2—C12—C13	108.72 (17)	N1—C15—C16	113.9 (2)
C14—C13—C12	110.37 (18)	N1—C15—H15A	108.8
C14—C13—Cl1	131.91 (17)	C16—C15—H15A	108.8
C12—C13—Cl1	117.71 (15)	N1—C15—H15B	108.8
O1—C11—O2	110.64 (16)	C16—C15—H15B	108.8
O1—C11—C14	108.76 (15)	H15A—C15—H15B	107.7
O2—C11—C14	105.02 (15)	C2—C3—C4	111.3 (2)
O1—C11—H11	110.8	C2—C3—C1	110.9 (2)
O2—C11—H11	110.8	C4—C3—C1	113.8 (2)
C14—C11—H11	110.8	C2—C3—H3	106.8
C9—C4—C3	113.48 (18)	C4—C3—H3	106.8
C9—C4—C5	108.78 (19)	C1—C3—H3	106.8
C3—C4—C5	113.70 (18)	C3—C2—H2A	109.5
C9—C4—H4	106.8	C3—C2—H2B	109.5
C3—C4—H4	106.8	H2A—C2—H2B	109.5
C5—C4—H4	106.8	C3—C2—H2C	109.5
C9—C8—C7	113.01 (18)	H2A—C2—H2C	109.5
C9—C8—H8A	109.0	H2B—C2—H2C	109.5
C7—C8—H8A	109.0	C6—C7—C10	112.5 (2)
C9—C8—H8B	109.0	C6—C7—C8	109.8 (2)
C7—C8—H8B	109.0	C10—C7—C8	110.6 (2)
H8A—C8—H8B	107.8	C6—C7—H7	107.9
O1—C9—C8	110.96 (15)	C10—C7—H7	107.9
O1—C9—C4	106.29 (16)	C8—C7—H7	107.9
C8—C9—C4	111.98 (17)	C16—C17—H17A	120.0
O1—C9—H9	109.2	C16—C17—H17B	120.0
C8—C9—H9	109.2	H17A—C17—H17B	120.0
C4—C9—H9	109.2	C17—C16—C15	126.5 (3)
C7—C6—C5	112.0 (2)	C17—C16—H16	116.8
C7—C6—H6A	109.2	C15—C16—H16	116.8
C5—C6—H6A	109.2	C3—C1—H1A	109.5
C7—C6—H6B	109.2	C3—C1—H1B	109.5
C5—C6—H6B	109.2	H1A—C1—H1B	109.5
H6A—C6—H6B	107.9	C3—C1—H1C	109.5
C20—C19—C18	125.4 (3)	H1A—C1—H1C	109.5
C20—C19—H19	117.3	H1B—C1—H1C	109.5
C18—C19—H19	117.3	C7—C10—H10A	109.5
C6—C5—C4	112.37 (19)	C7—C10—H10B	109.5
C6—C5—H5A	109.1	H10A—C10—H10B	109.5
C4—C5—H5A	109.1	C7—C10—H10C	109.5
C6—C5—H5B	109.1	H10A—C10—H10C	109.5
C4—C5—H5B	109.1	H10B—C10—H10C	109.5
H5A—C5—H5B	107.9	C11—O1—C9	115.87 (15)
N1—C18—C19	112.65 (18)	C12—O2—C11	109.26 (15)
N1—C18—H18A	109.1	C14—N1—C15	121.05 (18)
C19—C18—H18A	109.1	C14—N1—C18	123.58 (16)
N1—C18—H18B	109.1	C15—N1—C18	114.71 (17)
			
N1—C14—C13—C12	178.7 (2)	C9—C4—C3—C1	−69.3 (2)
C11—C14—C13—C12	−2.5 (2)	C5—C4—C3—C1	55.8 (3)
N1—C14—C13—Cl1	−0.1 (4)	C5—C6—C7—C10	−178.1 (2)
C11—C14—C13—Cl1	178.66 (18)	C5—C6—C7—C8	−54.5 (3)
O3—C12—C13—C14	179.4 (2)	C9—C8—C7—C6	54.1 (3)
O2—C12—C13—C14	−0.8 (2)	C9—C8—C7—C10	178.8 (2)
O3—C12—C13—Cl1	−1.6 (3)	N1—C15—C16—C17	−3.2 (4)
O2—C12—C13—Cl1	178.22 (15)	O2—C11—O1—C9	87.69 (19)
N1—C14—C11—O1	65.3 (2)	C14—C11—O1—C9	−157.46 (15)
C13—C14—C11—O1	−113.67 (19)	C8—C9—O1—C11	−68.4 (2)
N1—C14—C11—O2	−176.28 (18)	C4—C9—O1—C11	169.65 (16)
C13—C14—C11—O2	4.8 (2)	O3—C12—O2—C11	−176.1 (2)
C7—C8—C9—O1	−173.88 (18)	C13—C12—O2—C11	4.0 (2)
C7—C8—C9—C4	−55.3 (3)	O1—C11—O2—C12	111.83 (19)
C3—C4—C9—O1	−56.8 (2)	C14—C11—O2—C12	−5.3 (2)
C5—C4—C9—O1	175.58 (18)	C13—C14—N1—C15	12.0 (4)
C3—C4—C9—C8	−178.10 (18)	C11—C14—N1—C15	−166.6 (2)
C5—C4—C9—C8	54.3 (2)	C13—C14—N1—C18	−177.8 (2)
C7—C6—C5—C4	57.3 (3)	C11—C14—N1—C18	3.6 (3)
C9—C4—C5—C6	−55.5 (3)	C16—C15—N1—C14	79.1 (3)
C3—C4—C5—C6	177.0 (2)	C16—C15—N1—C18	−91.9 (3)
C20—C19—C18—N1	115.5 (3)	C19—C18—N1—C14	122.4 (2)
C9—C4—C3—C2	164.5 (2)	C19—C18—N1—C15	−66.8 (3)
C5—C4—C3—C2	−70.4 (3)		
